# Experience With Intermittent Caloric Restriction in Individuals With Type 1 Diabetes

**DOI:** 10.1155/jdr/3581401

**Published:** 2026-08-05

**Authors:** Alison Wong, Raul Herrera, Krista Varady, Qi Wang, Niki Oldenburg, Lisa S. Chow

**Affiliations:** ^1^ University of Minnesota, Minneapolis, Minnesota, USA, umn.edu; ^2^ Department of Medicine, Division of Diabetes, Endocrinology and Metabolism, University of Minnesota, Minneapolis, Minnesota, USA, umn.edu; ^3^ University of Illinois, Chicago, Illinois, USA, illinois.edu; ^4^ Clinical and Translational Science Institute, University of Minnesota, Minneapolis, Minnesota, USA, umn.edu; ^5^ Department of Medicine, Cardiovascular Division, University of Minnesota, Minneapolis, Minnesota, USA, umn.edu

## Abstract

**Background:**

Little is known about the prevalence and experiences of intermittent caloric restriction (ICR) among individuals with Type 1 diabetes mellitus (T1DM), despite its growing popularity.

**Methods and Results:**

From September to October 2023, patients diagnosed with T1DM aged 18–89 in Minnesota were invited via the M Health Fairview′s electronic health record (EHR) to complete a 10‐min online survey. The survey gathered patient‐provided data on demographics, clinical data, and questions centered on weight and use of ICR. Out of 124 respondents, 39 reported participating in ICR and completed the survey. These participants, with a mean age of 42.8 years (SD = 12.0) and a mean BMI of 28.2 kg/m^2^ (SD = 5.5), included 22 females and 17 males. Among them, 34 reported practicing time‐restricted eating (TRE), whereas 5 reported practicing intermittent fasting (IF). TRE participants reported an average weight loss of 6.0 kg, whereas IF participants reported an average weight loss of 3.5 kg. Hypoglycemia was reported by five TRE participants and one IF participant, with no episodes of diabetic ketoacidosis (DKA) reported. Only six participants reported that their healthcare providers were aware of their fasting program.

**Conclusions:**

Individuals with T1DM do engage in ICR, mainly TRE, with rare hypoglycemia and no DKA. Further research is needed on long‐term ICR safety in T1DM patients.

## 1. Introduction

In addition to lifestyle changes that have promoted increasing weight in the general population, advances in insulin treatment for individuals with Type 1 diabetes mellitus (T1DM) have also been associated with unintended metabolic consequences, including weight gain, insulin resistance, and the emergence of additional cardiovascular risk factors such as hypertension and dyslipidemia [1]. As a result, the prevalence of obesity is increasing in individuals with T1DM, with previously reported obesity rates of ~3.4% observed in patients with T1DM in the 1980s increasing to ~28%–37% in the 2020s [2].

Weight loss fundamentally requires a negative caloric balance, which can be achieved through various dietary strategies, including daily caloric restriction or intermittent caloric restriction (ICR) approaches such as intermittent fasting (IF) and time‐restricted eating (TRE). Although conventional weight management strategies have traditionally emphasized reducing caloric intake, IF and TRE focus on limiting the time window for eating. IF typically involves fasting every other day, 2 days per week, or several days per month [3], whereas TRE implements a daily 14–16‐h fasting window paired with an 8–10‐h eating window [4].

Although ICR has demonstrated efficacy in weight management for individuals without diabetes and those with Type 2 diabetes (T2DM), its application and potential effectiveness among patients with T1DM remain unexplored. There is a critical gap in understanding the lived experiences of T1DM patients who employ TRE for weight management, which limits our understanding of TRE′s feasibility in this population.

The purpose of this study was to evaluate the prevalence and lived experiences of ICR, including both IF and TRE, among individuals diagnosed with T1DM. Specifically, we are aimed at (1) determining the extent of IF/TRE adoption in this population, (2) characterizing participants′ experiences with this dietary approach, and (3) analyzing clinical outcomes, including adverse events such as hypoglycemia and diabetic ketoacidosis, weight management effectiveness, and patient‐reported satisfaction.

## 2. Research Design and Methods

This study was approved by the University of Minnesota Institutional Review Board.

Adults aged 18–89 years with T1DM within the M Health Fairview′s electronic health record (EHR) who resided in Minnesota were invited to participate in an anonymous survey between September and October 2023. The invitation included a link to a one‐time, 10‐minute survey using REDCap (Research Electronic Data Capture) hosted at the University of Minnesota. We identified patients aged 18–65 with T1DM who maintained active MyChart accounts. We excluded pregnant individuals, those with cognitive impairment, non‐Minnesota residents, and patients who had opted out of research participation. Invited patients provided informed consent before completing a screening survey for pregnancy status and fasting program engagement. Only nonpregnant individuals who reported participating in fasting programs proceeded to the full questionnaire. We classified participants as practicing IF if they reported eating normally most days but consuming very low or no food on specific days. Those who reported maintaining a daily eating window were classified as TRE practitioners.

The survey included questions regarding demographics, weight loss goals, weight loss medication use, sleep patterns, meal habits, program satisfaction, and fasting‐related challenges. The survey also collected information about diabetes management, including glucose monitoring methods, insulin delivery systems, and fasting‐related insulin adjustments. With respect to adverse events during fasting, we asked about episodes of diabetic ketoacidosis during fasting and hypoglycemias requiring assistance, which is a proxy for severe hypoglycemia. No data were obtained from the medical record.

## 3. Results

Of the 124 patients who responded to the invitation to participate, 39 (31.5%) reported engaging in a fasting program and completed the survey. Only 6 (15.38%) reported that their primary provider was aware of their participating in a fasting program.

### 3.1. Demographics

There were 22 (56%) female respondents and 17 (44%) male respondents. The age (mean ± SD) was 42.8 ± 12.0 years, and the mean age of T1DM diagnosis was 20.8 ± 12.2 years. The mean weight was 83.5 ± 17.7 kg with a mean BMI of 28.2 ± 5.5 kg/m^2^.

### 3.2. Fasting/Dietary Habits

As reported in Table 1, of the 39 patients who reported participating in a fasting program, 34 (87%) reported engaging in TRE, and 5 (13%) reported engaging in IF. The median duration of the fasting program was 22 months (IQR, 9.5–58). The mean eating window for TRE patients was 8.5 ± 2.6 h with a mean start time at 10 : 30 ± 3.6 h and a mean end time at 19 : 43 ± 3.2 h. During their fasting period, the TRE group reported consumed water (*n* = 29), coffee (*n* = 18), noncaloric drinks (*n* = 18), and/or less food than usual (*n* = 17) in their diet. During the fasting period, the IF group reported either fasting or very low intake for an average of 2.2 days each week. The IF participants reported consuming water, coffee, and noncaloric drinks during their fasting period.

**Table 1:** Survey results.

	All (*n* = 39)	Intermittent fasting (*n* = 5)	Time restricted eating (*n* = 34)
Demographics, mean (SD)			
Age at diagnosis, years	20.8 ± 12.2	26.6 ± 15.7	19.9 ± 11.5
Current age, years	42.8 ± 12.0	39.8 ± 4.5	43.2 ± 12.6
Diabetes duration, years, mean (SD)	22 ± 13	13 ± 11	22.7 ± 13
Weight, kg	83.5 ± 17.7	80.2 ± 22.7	83.9 ± 16.9
BMI, kg/m^2^	28.2 ± 5.5	27.5 ± 9.3	28.3 ± 4.8
Weight loss, *n* (%)			
Weight loss without medication	34 (87.2)	5 (100)	29 (85.2)
Weight loss with medication	5 (12.8)	0	5 (14.7)
Weight loss, kg	5.9 ± 6.4	3.5 ± 3.4	6.3 ± 6.5
Weight loss medication, *n* (%)			
GLP‐1a	4 (10.3)	0	4 (11.8)
Metformin	1 (2.6)	0	1 (2.9)
Complications, *n* (%)			
Hypoglycemia requiring assistance in last year	6 (15.3)	1 (20)	5 (14.7)
Hypoglycemia related to fasting program	2 (33.3%)	1 (100)	1 (20%)
Hypoglycemia required assistance	5 (83.3%)	0	5 (83.3%)
DKA	0	0	0
CGM use, *n* (%)	38 (97.4)	5 (100)	33 (97.1)
Treatment, *n* (%)			
MDI	9 (23.1)	2 (40)	7 (20.6)
Insulin pump	30 (76.9)	3 (60)	27 (79.4)
Types, *n* (%)			
Tandem t:slim	15 (50)	1 (33.3)	14 (51.8)
OmniPod 5	9 (33.3)	1 (33.3)	8 (26.6)
Medtronic	4 (14.8)	1 (33.3)	3 (11.1)
OmniPod DASH	2 (6)		
Other			2 (7.4)
Reported need to adjust insulin in response to fasting program	14 (35.9)	1 (20)	13 (38.2)
Types of changes			
Decrease in basal insulin	9 (23)	1 (20)	8 (23.5)
Decrease in prandial insulin	2 (5.1)	0	2 (5.8)
Other	2 (5.1)	1 (20)	3 (8.8)
Satisfaction with fasting program, *n* (%)			
Willing to recommend to friends and family	26 (66.7)	3 (60)	23 (67.6)
Willing to recommend to other patients with T1DM	29 (74.4)	2 (40)	27 (79.4)

*Note:* Values are presented as mean ± standard deviation (SD) or number (%).

Abbreviations: CGM, continuous glucose monitor; DKA, diabetic ketoacidosis; T1DM, Type 1 diabetes mellitus.

### 3.3. Weight Loss

Participants in the TRE group reported an average weight loss of 6.3 kg over the past year, whereas those in the IF group reported an average weight loss of 3.5 kg. Five patients in the TRE group were also using medications associated with weight loss such as semaglutide (3), liraglutide (1), or metformin (1), whereas none in the IF group reported taking medications for weight loss.

### 3.4. Complications

As shown in Table 1, hypoglycemic events occurred in one IF participant and five TRE patients. Only two TRE participants attributed their hypoglycemia to fasting. No participants experienced DKA.

### 3.5. Glucose Monitoring and Insulin Delivery

As shown in Table 1, 38 (97%) participants used CGMs, and 30 (77%) used insulin pumps, with 27 (90%) being hybrid closed‐loop systems. The Tandem t:slim was most common (15 participants, 50%). Fourteen participants (35.9%) adjusted their insulin in response to their fasting program, primarily by reducing basal rates.

### 3.6. Satisfaction With Fasting Program

As shown in Table 1, 26 participants (66.7%) would recommend their fasting program to friends and family, and 29 (75%) would recommend it to others with T1DM.

## 4. Discussion

The main finding of this study is that one‐third of T1DM patients practiced ICR, with no reported DKA episodes. Although some experienced hypoglycemia, most did not attribute it to ICR. Most participants used advanced technology (75% hybrid closed‐loop pumps, 97% CGM), and one‐third modified insulin doses for ICR. With 75% willing to recommend ICR to others with T1DM, findings suggest ICR may be feasible when supported by glucose monitoring and insulin adjustment protocols.

These findings extend the current literature of ICR in T1DM. Several studies have examined the short‐term effects (≤ 7 days) on fasting in T1DM. [5, 6]. These studies report that the rate of hypoglycemia is low with fasting and not clearly increased with more prolonged fasting (7% in a 12‐h fast and 6% in a 36‐h fast). During 36 h of fasting, there was a mild elevation in ketones; no participants developed DKA [5]. In a study of 19 patients with T1DM completing a 7‐day fast with daily basal insulin reductions, no severe hypoglycemia or DKA occurred [6]. Our results extend these findings, reporting rare DKA and good tolerance in patients with T1DM who report a prolonged history of engaging in ICR. It is important to recognize that a mild elevation in ketones can be expected during fasting, but for an individual to develop DKA, there also needs to be insulin deficiency, making it important that individuals who are practicing ICR need to be counseled on the fact that although their insulin requirements may decrease, and they will need lower doses to lower their risk of hypoglycemia, they still need to receive sufficient insulin to prevent ketoacidosis.

Although research on ICR is limited for T1DM, studies have established ICR′s effectiveness for weight loss (~5%) in people without diabetes and those with T2DM [7, 8], albeit individuals without diabetes or with T2DM have a lower risk for hypoglycemia than patients with T1DM. As noted in the study, the majority of patients with T1DM who participate in fasting wear CGM as well as an insulin pump, suggesting that technology may increase the comfort level for participating in ICR as well as reducing hypoglycemia risk [9].

This research expands current literature by identifying a critical communication gap between providers and individuals with T1DM regarding fasting practices. Most participants engaged in IF without their healthcare providers′ knowledge, a disconnect that may hinder proactive insulin management and increase metabolic risks. The American Diabetes Association recommends that healthcare providers should know if individuals with diabetes treated with insulin practice religious or nonreligious fasting and if so that they undergo prefast risk assessment in order to reduce the risks of fasting [10]. Inquiring about current and ideally anticipated changes to lifestyle habits including nutrition and physical activity, should be part of the visit for any individual with diabetes, no different from the reconciliation of current insulin doses. Notably, most participants engaging in ICR reported they were likely to recommend ICR to others with T1DM. Previous research has shown that adolescents and young adults with T1DM value peer support for diabetes self‐management [11]. Thus, positive experiences with ICR may promote wider adoption of these dietary programs through peer networks.

The strength of this study is in its examination of fasting habits in patients with T1DM, documenting weight loss goals, medication use, meal schedules, program satisfaction, and fasting‐related challenges.

There are several limitations to this study. First, our reliance on self‐reported data introduces recall bias with respect to weight changes, specifics on time restricted eating, frequency of hypoglycemia management, and insulin use prior to and after dietary interventions. Second, we did not obtain data from the medical record such as hemoglobin A1c or continuous glucose monitoring, information which would be useful for developing clinical guidance. Third, our study population was small and restricted to one healthcare system, which would limit application of our findings to other populations. The self‐reported data limitation could be addressed in future studies by data validation against the electronic medical record as well as obtaining glycemic control data such as hemoglobin A1c and continuous glucose monitor and insulin pump reports where available. Additional questions to better understand dietary practices and their motivation for them could provide further insights. A larger sample size would allow for results to be not only more robust, but also more generalizable to other populations.

## 5. Conclusion

This study supports that patients with T1DM engage in fasting programs with high satisfaction while reporting few adverse events. Given the rising prevalence of obesity and cardiovascular risk factors in T1DM patients, future research could investigate the effects of long‐term fasting on insulin use, weight, and cardiovascular outcomes. This research would help healthcare providers develop optimal insulin management protocols and evidence‐based guidance for patients with T1DM interested in fasting.

## Author Contributions

Alison Wong and Raul Herrera are co‐first authors.

## Funding

No funding was received for this manuscript.

## Disclosure

The authors have nothing to report.

## Conflicts of Interest

The authors declare no conflicts of interest.

## Supporting information


**Supporting Information** Additional supporting information can be found online in the Supporting Information section. Supporting Information. File S1: Survey used to collect patient reported data including demographics, fasting habits, and Type 1 diabetes management.

## Data Availability

The data that support the findings of this study are available from the corresponding authors upon reasonable request.
