# Supplementary material for: Experience With Intermittent Caloric Restriction in Individuals With Type 1 Diabetes
Source: J Diabetes Res. 2026 Aug 5;2026:3581401. doi: 10.1155/jdr/3581401 (PMC13443113; doi:10.1155/jdr/3581401)
Supplement: Supplementary file 1 [file JDR-2026-3581401-s001.pdf]

Survey of Fasting Habits in T1DM

You have been identified as a patient with Type 1 diabetes (T1DM) in the EHR who has given consent to be contacted about research studies.

The purpose of this survey is to guide the UMN Diabetes, Endocrinology and Metabolism (DEM) division to explore whether fasting might be a good intervention for patients with T1DM. As such, we are curious if patients with T1DM already engage in fasting as part of their usual lifestyle habits.

Fasting is typically described as one of 2 options :

1) Intermittent fasting: Eating normally for several days per week and then eating very little for remaining days per week.

or

2) Time Restricted Eating: You have designated an eating window during which you eat normally and outside the window, you would "fast."

If you are interested, please fill this survey which will take about 10 minutes.

Thank you!

UMN DEM division

Are you currently participating in a fasting program (either intermittent fasting OR time-restricted eating)?

○ Yes
○ No

Fasting Program:
Intermittent Fasting: Eating normally for several days per week and then eating very little for remaining days per week.

OR
Time Restricted Eating: You have designated an eating window during which you eat normally and outside the window, you would "fast."

Are you currently pregnant?

○ Yes
○ No
○ Not applicable

Thank you for volunteering to answer our survey. It should take about 10 minutes or less to complete.

Please be sure to press the SUBMIT button at the bottom of the survey when you have completed the questions.

We will start by asking a few general demographic and health questions.

At what age were you diagnosed with Type I Diabetes?

____

Please report your height in inches.	______________________________
Please report your weight in pounds.	______________________________
Please report your age.	______________________________ (years)
Please report your sex.	◯ Male<br>◯ Female<br>◯ Other<br>◯ Prefer not to say
If other, please describe	______________________________
Are you currently actively trying to lose weight?	◯ Yes<br>◯ No
How much weight in pounds have you lost over the past year?	______________________________
Are you currently on any medications which specialize in weight loss (examples include Ozempic, Trulicity, Bydureon, Mounjaro, Victoza, Byetta or Phenteramine)?	◯ Yes<br>◯ No
What are you taking?	◯ Ozempic<br>◯ Trulicity<br>◯ Mounjaro<br>◯ Bydureon<br>◯ Victoza<br>◯ Byetta<br>◯ Other/don't know
If you noted "Other or Don't know" - please describe	______________________________
What time do you typically go to bed during the weekday?	______________________________
What time do you typically go to bed during the weekend?	______________________________
What time do you typically wake up during the weekday?	______________________________
What time do you typically wake up during the weekend?	______________________________

Now we wish to ask you a few questions about your Fasting Program

Approximately when did you start your fasting program? (Report month and year- ie January 2021) ________________________________

Is your primary provider aware that you are participating in a fasting program?
- ◯ Yes
- ◯ No
- ◯ Don't Know

If your provider is aware that you are participating in a fasting program, are they supervising you for complications related to the fasting program?
- ◯ Yes
- ◯ No
- ◯ Don't Know

Are you in a fasting program where you would eat normally for several days per week, and either no intake or very low intake for the remaining days of the week (intermittent fasting)?
- ◯ Yes
- ◯ No

If so, how many days per week do you eat normally?
- ◯ 0
- ◯ 1
- ◯ 2
- ◯ 3
- ◯ 4
- ◯ 5
- ◯ 6
- ◯ 7

How many days per week do you either fast or have very low intake?
- ◯ 0
- ◯ 1
- ◯ 2
- ◯ 3
- ◯ 4
- ◯ 5
- ◯ 6
- ◯ 7

On the days that you fast, what do you eat if anything? Check all that apply
- ☐ Water
- ☐ Coffee
- ☐ Noncaloric drinks
- ☐ Food (smaller amount than usual)
- ☐ Other

If you said other, please describe ________________________________

Are you in a fasting program where you have an eating window every day (time restricted eating)?
- ◯ Yes
- ◯ No

How long is your daily eating window (hours)
- ◯ < 4 hours
- ◯ 4 hours
- ◯ 5 hours
- ◯ 6 hours
- ◯ 7 hours
- ◯ 8 hours
- ◯ 9 hours
- ◯ 10 hours
- ◯ 11 hours
- ◯ 12 hours
- ◯ other

If you stated "other" - please describe

____

On average, what time does your eating window start?

____

On average, what time does your eating window stop?

____

On the days that you fast, what do you eat if anything? Check all that apply

- ☐ Water
- ☐ Coffee
- ☐ Noncaloric drinks
- ☐ Food (smaller amount than usual)
- ☐ Other

If you said "other" - please describe

____

Acceptability of Fasting Program

Would you recommend your fasting program to your friends and family?

- ◯ Yes
- ◯ No

Do you believe that your fasting program is dangerous with regards to your glycemic control?

- ◯ Yes
- ◯ No

Would you recommend your fasting program to other patients with Type I Diabetes?

- ◯ Yes
- ◯ No

Complications of Fasting Program

Have you had any complications related to your fasting program?

- ◯ Yes
- ◯ No

Please explain.

____

Have you had any episodes of Diabetic Ketoacidosis within the past year which required hospitalization? (Which may or may not be related to your fasting program)

- ◯ Yes
- ◯ No

Have you had any problems with Diabetic Ketoacidosis related to your fasting program?

- ◯ Yes
- ◯ No

Number of DKA episodes within the past year which required either an ER visit or hospitalization?

- ◯ 1
- ◯ 2
- ◯ 3
- ◯ 4
- ◯ 5
- ◯ 6
- ◯ 7+

Have you had any episodes of Hypoglycemia within the past year which required assistance of another person? (Which may or may not be related to your fasting program)	◯ Yes<br>◯ No
Number of hypoglycemia episodes within the past year which required the assistance of another person	◯ 1<br>◯ 2<br>◯ 3<br>◯ 4<br>◯ 5<br>◯ 6<br>◯ 7+
Have you had any problems with Hypoglycemia related to your fasting program?	◯ Yes<br>◯ No
Please explain.	__________________________________
Have you had to adjust your insulin program because of your fasting program?	◯ Yes<br>◯ No
If you had to adjust your insulin program because of your fasting program, what did you do?	__________________________________

You are almost done with the survey.

The next few questions address how you manage your Type 1 Diabetes.

Are you currently using an insulin pump?	◯ Yes<br>◯ No
Is your insulin pump a closed-loop pump (Tandem T-Slim or Omnipod 5 or Medtronic pump)?	◯ Yes<br>◯ No<br>◯ Unsure
Please describe the type of your closed loop pump	◯ Tandem t-slim<br>◯ Omnipod5<br>◯ Medtronic
If you are not using a closed loop pump or unsure if you are using a closed loop pump, please indicate the name and model of your pump	____________________________
What percent of the time do you use the closed-loop feature?	0% 50% 100%<br>*(Place a mark on the scale above)*
Are you currently routinely using insulin injections by either vial/syringe or pens?	◯ Yes<br>◯ No
Are you currently using a continuous glucose monitor?	◯ Yes<br>◯ No

If yes, what brand?	◯ Dexcom (any version)<br>◯ Libre (any version)<br>◯ Medtronic (any version)<br>◯ Unknown/Unsure
If you are unsure, please indicate the name and model of your CGM	__________________________________
